# Current status of the immunogenicity of enzyme replacement therapy in fabry disease

**DOI:** 10.1186/s13023-025-03705-4

**Published:** 2025-05-26

**Authors:** Jorge F. Gómez-Cerezo, Julián Fernández-Martín, Miguel Ángel Barba-Romero, Rosario Sánchez-Martínez, Alvaro Hermida-Ameijeiras, Maria Camprodon-Gómez, Saida Ortolano, Mónica A. Lopez-Rodriguez

**Affiliations:** 1 European University of Madrid Biomedical Research Foundation, Infanta Sofia University Hospital and Henares University Hospital, Madrid, Spain; 2 Working Group on Rare Diseases of the Spanish Society of Internal Medicine (GTEM-SEMI), Madrid, Spain; 3 Internal Medicine Department, Complejo Hospitalario Universitario de Vigo, 36312 Vigo, Spain; 4 Rare Diseases & Pediatric Medicine Research Group, Galicia Sur Health Research Institute (IIS Galicia Sur), SERGAS-UVIGO, Vigo, Spain; 5 Department of Internal Medicine, Hospital General Universitario de Albacete, Albacete, Spain; 6 Internal Medicine Department, Alicante General University Hospital-Alicante Institute of Health and Biomedical Research (ISABIAL), Pintor Baeza 12, 03010 Alicante, Spain; 7 Division of Internal Medicine, UETeM-Molecular Pathology Group, MetabERN, IDIS-CIMUS, University of Santiago de Compostela, Santiago de Compostela, Spain; 8 Department of Internal Medicine, Unit of Hereditary Metabolic Disorders, Vall d’Hebron University Hospital, Passeig Vall d’Hebron 119-129, 08035 Barcelona, Spain; 9 Faculty of Medicine and Health Sciences, Universidad de Alcalá (UAH), Alcalá de Henares, 28871 Av. de Madrid, Spain

**Keywords:** Fabry, Immunogenicity, Antidrug antibodies, Enzyme replacement therapy, Agalsidase, Pegunigalsidase

## Abstract

In patients with Fabry disease (FD), treatment with enzyme replacement therapy (ERT), may trigger the formation of anti-drug antibodies (ADAs). The consequences of this immune reaction range from the transient appearance of clinically insignificant ADAs to the generation of neutralizing antibodies that negate the clinical benefit of the biotherapeutic agent, lead to side effects (such as injection site reactions), and even cause severe, life-threatening symptoms. Many factors may influence the immunogenicity of these therapeutic proteins. Currently, there are three commercially available long-term ERT treatments in patients with FD: agalsidase alfa, agalsidase beta, and more recently, pegunigalsidase alfa. Neutralizing ADAs are present in approximately 40% of male FD patients treated with ERT based on agalsidase alfa or agalsidase beta and have shown in vitro cross-reactivity with both agalsidases. Their formation seems to be irreversible, meaning that most patients with positive neutralizing ADAs remain so for up to 10 years after starting treatment. Recent studies show that in some patients, pre-existing ADAs against agalsidase alfa and agalsidase beta have lower affinity and lower inhibitory effects against pegunigalsidase alfa. Additionally, in clinical trials involving naïve patients, neutralizing antibodies were mostly transient, although further studies are needed to confirm these findings in clinical practice. The formation of ADAs is often associated with a worse clinical prognosis and a faster progression of the disease. Given the rapid progression of FD, measuring ADAs titers is essential to provide personalised treatment for each patient. This is why international recommendations highlight the importance of monitoring the existence of ADAs, their neutralising activity, and globotriaosylsphingosine (lyso-Gb3) levels in patients receiving ERT. However, several unresolved issues remain, such as the importance of ADAs levels (particularly neutralising ADAs), the standardisation of assay methods, the interpretation of results, and the implications of these findings for therapeutic strategies. Overcoming the development of ADAs is critical to improving treatment outcomes in patients with FD, different strategies have been explored to address this challenge. The present work aims to review latest developments related to all aspects mentioned above, while also analyzing the potential role of therapeutic innovations.

## Background

This article reviews different aspects of immunogenicity and its impact on the generation of neutralizing antibodies, which may compromise the efficacy of ERT in FD. It is well-established that the formation of ADAs is often associated with a worse clinical prognosis and accelerated disease progression. However, several unresolved issues remain, such as the importance of ADAs levels, the standardization of assay methods, the interpretation of results and the implications of these findings for therapeutic strategies. Additionally, this article examines newly published evidence as well as the recent approval of new treatments with immunogenic profiles that differ from those observed with standard therapy. A better understanding of immunogenicity is crucial to enhancing our ability to effectively treat patients with this debilitating disease.

## Main text

## Fabry disease: definition and prevalence

FD is an inherited lysosomal storage disorder linked to the X chromosome [1, 2] caused by pathogenic variants in the galactosidase alpha (*GLA*) gene (Xq21.3-q22), leading to an absence or deficiency of alpha-galactosidase A enzyme (α-Gal A) [3]. Such deficiency leds to the progressive build-up mainly of non-metabolised globotriaosylceramide (Gb3) and its deacylated derivative, lyso-Gb3, in most types of cells and bodily fluids [4]. Whithin cells, Gb3 and, mainly, lyso-Gb3 behave as a causal factor of tissue injury, particularly affecting the heart, kidneys, central and peripheral nervous system, skin and eyes [5]. Moreover, lyso-Gb3 is used as a disease biomarker, although its interpretation depends on the phenotype and gender [1].

FD can be classified in two phenotypes—classic and late onset. The clinical features of these two genotypes have been extensively described in multiple review articles [6–11]. Clinical trial-based estimations of FD worldwide prevalence range between 1:40,000 and 1:170,000 [9].

Current pharmacological treatment includes ERT, and the use of pharmacological chaperones (e.g. migalastat) to stabilize some mutated enzymes, together with support treatment whenever it is appropriate [3].

The main goal of FD treatment is to revert the symptoms and prevent progression in most domains associated to the disease, thereby improving patients’ health-related quality of life (HRQoL) [12].

For over two decades, ERT obtained through recombinant DNA technology has provided the opportunity to slow disease progression and even achieve remission of some clinical features, with a potentially improved prognosis in the long term [13]. Both classic and late onset FD patients require treatment to prevent disease progression. There is increasing evidence that an early start of ERT optimizes treatment benefits and can prevent or delay disease progression [12].

ERT comprises intravenous perfusion, every other week, of the deficient enzyme [14–16].

## Introduction to immunogenicity of therapeutic proteins

Recombinant therapeutic proteins are recognized by the human immune system and usually trigger a complex, potentially harmful immune response, with the formation of ADAs, T-cell activation, and innate immune responses [17].

Upon observation of the functional impact of ADAs, two types may be distinguished: Non-neutralizing ADAs (or binding antibodies, BAbs), that specifically bind with the drug, but do not affect the drug-substrate interaction; and neutralizing ADAs (NAbs), which directly bind with—or are very close to—the pharmacologically active site of the drug, and physically interfere with its ability to bind to its target. While BAbs may indirectly decrease the drug level as they increase its suppression through the formation of immune complexes (ICs), NAbs may have a direct negative impact at the drug’s functional level [18]. NAbs may entail a safety issue for enzyme replacement therapies, where cross-reactivity and the subsequent neutralization of the endogenous counterpart may bring about side effects [19].

The consequences of an immune reaction to a therapeutic protein range from the transient occurrence of ADAs with no clinical significance to severe, life-threatening affections [17]. Many factors related to the patient, the condition and the product can have an influence on the immunogenicity of therapeutic proteins. ADAs can have an impact on the efficacy of a therapeutic protein, either interfering with the pharmacodynamic interaction between the therapeutic protein and the substrate or altering its pharmacokinetics (PK) profile [17]. Although in general most adverse effects of therapeutic proteins are related to their pharmacological effect, the main exception are immune reactions, as they can cause both acute and delayed adverse effects [17].

In FD, ERT involves intravenous infusion of α-Gal A protein, which may cause a humoral immune response and generate ADAs, considering it is administered in multiple doses for prolonged periods [20, 21]. Humoral immune response is typically observed in males whit the classic phenotype of FD, who have little or no native enzyme, which means that the immune system recognizes the exogenously administered enzyme as strange, as there has been no previous contact with the native protein during development [22–26]. More often in males than females, therapeutic proteins can be recognized as strange to the body, and consequently, they can cause infusion-related reactions (IRRs) and the formation of NAbs, which involve a decrease in treatment efficacy and disease progression [27].

Immunoglobulin E (IgE)-mediated response is scarcely reported in patients treated with ERT, which suggests that IRRs are independent of IgE and, therefore, they mostly result from anaphylactoid reactions rather than anaphylactic reactions [20]. Patients with ADAs against agalsidase are at higher risk of suffering IRRs [20].

Currently, there are three commercially available treatments as a long-term ERT in patients with a confirmed diagnosis of FD: agalsidase alfa (Replagal®, Takeda) produced in a human-origin cell line (human fibrosarcoma cells HT-1080), at approved dose of 0.2 mg/kg every 2 weeks [14]; agalsidase beta (Fabrazyme®, Sanofi), produced in Chinese hamster ovary (CHO) cells at approved for a 1 mg/kg dose every 2 weeks [15], and pegunigalsidasealfa (, Elfabrio®, Chiesi), produced in tobacco cells and approved dose of 1 mg/kg every 2 weeks [16]. According to each summary of product characteristics, most patients treated with agalsidase beta develop ADAs against the enzyme [15], whereas those were observed in 24% of males treated with agalsidase alfa [14]. Recent comparative studies have shown a significantly higher risk of developing ADAs in patients treated with agalsidase beta [28]. This difference may be due to different approved doses for every product, to the different cell lines where they are produced, and the CRIM (*Cross Reactive Immunological Material*) status of treated patients, although the latter factor has only recently been considered [29]. Pegunigalsidasealfa is a pegylated, covalently reticulated form of α-Gal A [30–32]. Preliminary studies on pegunigalsidasealfa have suggested a lower immunogenicity in comparison with agalsidase-alfa and beta [32]. This effect may be caused by the prolonged stability and increased half life of the enzyme in plasma, due to pegylation and stable reticulated homodimerization. It has also been suggested that some epitopes may be masked by pegylation [33]. A reduced immunogenicity may lead to a better therapeutic effect in male patients with classic phenotype treated with pegunigalsidasealfa who have never received ERT and, potentially, also in patients already treated with pre-existing ADAs, due to an absent immune response (anergy) in patients who have never received ERT, or to a tolerance induction, respectively [33]. According to the summary of product characteristics, in clinical trials with pegunigalsidase, 16% of patients treated with 1 mg/kg every 2 weeks and 0% of patients treated with 2 mg/kg every 4 weeks developed treatment-induced ADAs [16].

## Fabry disease: ERT and anti-drug antibodies (ADAs)

### ADAs prevalence in FD patients under ERT treatment

Circulating ADAs may alter the pharmacokinetic (PK) and pharmacodynamic properties (PD) of a drug, reducing its efficacy. Depending on their titers, there may be a clinically meaningful reduction in the drug concentration Patients with low ADA titers drug concentrations enough to be effective can remain, whereas in patients with high titers, a relevant portion of the drug is neutralized and is likely to produce a lack of clinical response over time [34–36].

NAbs develop in approximately 40% of all FD male patients treated with ERT based on agalsidase alfa or agalsidase beta [37, 38] and have shown cross-reactivity in vitro with both molecules [39].

ADAs appear within the first 3 to 6 months after initiating ERT [37, 39], and in most patients with NAbs remain detectable up to 10 years after initiating the treatment [28, 38, 40]. The high prevalence of antibodies is probably because most FD male patients do not express residual enzyme activity [39]. According to Mauhin and colleagues, antibody production is more likely in males with classic phenotypes, regardless of the agalsidase molecule administered (alfa or beta). Moreover, those patients maintain higher lyso-Gb3 levels suggesting that antibodies are associated to a more severe condition [41].

In the study by Linthorst and colleagues [39], patients with IgG-positive sera assessed with ELISA showed a marked enzyme activity inhibition in vitro, ranging between 65 and 95%. No differences were observed in the inhibitor effect of IgG ADAs between patients treated with both agalsidases [39].

When considering the type of agalsidase (alfa or beta), used at their licensed doses, the existence of ADAs has been reported in 73%, to 91% of patients treated with agalsidase beta [26, 42, 43], and in 20% to 55% of males treated with agalsidase alfa [44, 45]. Although no significant differences have been shown with the use of the same dose (0.2 mg/kg every 2 weeks) at the beginning of ERT for both drugs [40].

With regard to pegunigalsidase alfa, in the clinical trial by Schiffmann and colleagues with pegunigalsidase alfa administered at increasing doses of 0.2 mg/kg, 1.0 mg/kg, or 2.0 mg/kg by intravenous infusion every two weeks for one year, in adult FD patients who had never received ERT, a relatively low immunogenicity was observed, and only 3 patients (17%) developed IgG ADAs [32, 46]. All the three patients turned negative after one year of treatment, which suggests immune tolerance induction (ITI). In two patients (11%), ADAs were temporarily positive for a neutralizing activity which disappeared, as the treatment advanced [32].

In the BRIDGE study [4], conducted in patients previously treated with agalsidase alfa and switched to pegunigalsidase alfa, 7 out of 20 patients (35%) showed positive results for anti-pegunigalsidase alfa IgG ADAs at ≥ 1 time during the study [4]. Of those, 2 had pre-existing ADAs at baseline (both males), and 5 (3 men, 2 women) were negative, subsequently developing anti-pegunigalsidase alfa IgG responses (induced responses). Among the 5 patients with ADA-induced responses, 3 had transient responses and eventually became ADA-negative, whereas 2 remained positive up to month 12. The 2 patients with pre-existing ADAs showed an increasing titer during the study, and were the only ones with neutralizing activity most of the time.

In the direct comparative study BALANCE [47], which compared pegunigalsidase alfa and agalsidase beta in patients with renal impairment previously treated with agalsidase beta for at least 1 year, treatment-emerging ADAs were present in 12% of patients treated with pegunigalsidasa alfa (6 out of 52; 3 ADA-negatives at the start of the treatment who turned positive during treatment, and 3 whose titers increased over four-fold during treatment). In the patients treated with agalsidase beta, treatment-emerging ADAs were present in 20% of them (5 out of 25 patients, 3 of which had treatment-induced ADAs, and 2 had increased titers).

The rate of total ADA-positive patients decreased slightly in both arms throughout the study period. However, the rate of patients with neutralizing antibodies decreased significantly with pegunigalsidase alfa (from 33 to 15%), compared to patients treated with agalsidase beta, where the percentage remained virtually the same (from 28 to 26%).

Baseline reactivity to pegunigalsidase alfa is explained by cross-reactivity with the aminoacid sequence shared by pegunigalsidase alfa and agalsidase beta. Recent studies have shown that, in some patients, ADAs against agalsidase alfa and agalsidase beta have lower affinity and less enzyme inhibiting effects against pegunigalsidase alfa [33]. Nevertheless, it is currently not possible to predict which ADA-positive patients may benefit from the change in ERT. Further studies are required, including real-world immunogenicity profile of pegunigalsidase alfa [46].

In a recent study by Lenders [33] and colleagues, cross-reactivity of ADAs against agalsidase alfa and agalsidase beta was analyzed in 49 FD patients, compared to pegunigalsidase alfa. The affinity of anti-alpha galactosidase antibodies in pooled patient sera was significantly lower for pegunigalsidase alfa than for agalsidase alfa and agalsidase beta. Protein pull-down precipitation tests revealed the presence of epitopes masked in pegunigalsidase alfa, possibly due to pegylation. Besides, the affinities of ADAs in patients against pegunigalsidase alfa (K_d_: 3.55 ± 2.72 mmol) were significantly lower than with agalsidase alfa (Kd: 1.99 ± 1.26 mmol) and agalsidase beta (K_d_: 2.18 ± 1.51 mmol) (p < 0.0001 in both cases). Inhibition analyses also revealed a 30% reduction in the inhibitory capacity of pre-existing ADAs towards pegunigalsidase alfa. Enzyme uptake experiments in AGAL-deficient EA.hy926 cells proved less ADA effects in cell uptake of pegunigalsidase alfa compared to agalsidase beta. The study suggests that, given the reduced affinity toward pre-existing ADAs to pegunigalsidase alfa, patients with ADAs against agalsidase alfa or beta may benefit from switching to pegunigalsidase alfa.

Recently, Bernat and colleagues [48] presented results from their study analysing the appearance of ADAs, both de novo and pre-existing, based on the clinical trial program with pegunigalsidase alfa. The study program includes both patients treated with other ERTs switched to pegunigalsidase alfa and *naïve* patients. According to the authors, a low incidence of de novo ADAs was observed, which were neutralizing in less than 25% of cases, compared to almost all patients that were ADA-positive at the beginning of the study, which suggests that de novo ADAs with pegunigalsidase alfa are less likely to be neutralizing compared to those developed with other ERTs. Besides, patients with de novo ADAs generally presented low titers, and were more likely to turn negative by the end of the study, compared to patients with pre-existing ADAs.

### ADAs levels and neutralizing activity

Given the rapid progression of FD, measuring ADAs titers is fundamental to provide a custom treatment to each patient [49]. Therefore, assessing and reporting on the immunogenicity of biotherapeutic products, including the titers of NAbs is a regulatory requirement during the clinical development of the drug, linked to the immunogenicity risk assessment [50].

With detection assays becoming increasingly sensitive to the drugs used today, the reliable estimation of the ADAs titer has gained significance to understand clinically relevant ADAs levels [51]. Given their influence in the accuracy of the resulting titer, the choice of a cut point factor of the titer deserves careful consideration [51]. Recently, Zhang and colleagues (2023) have proposed a method that may be widely applicable to immunogenicity assays. According to the authors, this method allows for a simplified implementation and administration and provides a sound statistical base to improve titer estimations [51].

#### Assessment of neutralizing activity

Criteria for evaluating ADAs detection assays that are typically recommended and used in clinical practice, as well as criteria for confirmatory assays, have been described in many previous publications [17, 51–54].

Some studies have used animal-sourced anti-α-Gal A antibodies as positive controls [41]. However, given the different species, different secondary antibodies are required for the final detection, which does not allow an appropriate quantification. Besides, animal-sourced polyclonal antibodies may differ in their individual binding affinity and epitope recognition compared to human antibodies, and so they are not recommended as appropriate controls for human ADAs quantification [27]. Thus, the use of a human reference antibody with a known concentration and comparable biochemical characteristics for the quantification of ADAs titers in human samples is strongly advised [27]. To overcome the limitations, van der Veen and colleagues pooled sera from FD patients with ADAs to generate a positive control, allowing ADAs measurements with the same secondary detection antibodies [55]. However, due to the unknown antibody concentration within the positive control, even this approach did not allow a quantification of measured ADAs titers. Lenders and colleagues (2021) generated a polyclonal reference antibody by pooling purified and characterized anti-AGAL antibodies from 22 patients, allowing an ELISA-based measurement and ADAs titer quantification expressed as ng/mL serum [49]. The use of ELISA-based assays also allows for the determination and differentiation of appropriate IgG isotypes [27].

Given that high affinity for ADAs is seemingly associated to increasing inhibitory abilities [56], whereas a decreasing affinity may point at the start of tolerance induction [57], performing the appropriate analyses is crucial for identify FD patients at risk [27].

#### Assessment of NAbs titers

In the assessment of neutralizing activity of the ADAs, it is important to consider the quasi-quantitative (if titer is reported) or qualitative (positive/negative) nature of the results, as there is no authentic calibration reference standard and surrogate positive control(s) are used to characterize the method. Therefore, clinical relevance must be established through careful analysis of the results in the context of their relationship to applicable clinical endpoints. [50].

In most cases, NAbs testing is conducted subsequent to ADAs testing, and testing in the NAbs assay is limited to ADA-positive (i.e., confirmed) samples for purposes of characterizing the immune response as either neutralizing (NAb-positive) or non-neutralizing (NAb-negative). There are many NAbs assay detection platforms currently in use, but all assays fall under two main categories: non-cell-based assays (i.e., ligand binding assays; LBAs) and cell-based assays. [50]. The overarching principle is that the type of assay should reflect the in vivo therapeutic mechanism of action (MoA) to generate clinically meaningful data [50].

### Analysis methods

ADA testing follows a tiered approach: a high-sensitivity screening assay detects ADAs, a confirmatory assay verifies specificity, and functional assays assess their impact (e.g., neutralizing activity, isotype, epitope). Screening methods include enzyme-linked immunosorbent assays (ELISA), electrochemiluminescence (MesoScale Diagnostics), microarrays (SQI Diagnostics), solid-phase assays (ImmunoCap), and bead-based assays (Gyros, Luminex). Less common techniques include surface plasmon resonance, radioimmunoprecipitation, and biolayer interferometry [58, 59].

For FD patients on ERT, ELISA-based measurments (including IgG subclass analyses) [39, 55], inhibitory measures, cell-based assays (to identify effects on cell ERT uptake) [60] and bedside tests [61] are commonly used. While all these methods can identify ADAs, titers are hardly comparable between assays or even between patient cohorts measured by different laboratories [49]. ELISA is favored for its reproducibility but lacks a human reference antibody, making absolute quantification challenging. Commercial antibodies have limitations, often recognizing only specific epitopes and requiring species-specific secondary antibodies [49]. In the absence of a reference, results are generally reported as relative values based on serial dilutions. A detection limit is previously set for the assay, based on absorbance values obtained from untreated samples (in the best-case scenario) or control samples (most common).

As previously comented, NAbs detection methods fall into non-cell-based and cell-based assays [50]. Regulatory agencies prefer cell-based assays for high-risk biologics, as they may better reflect the biological activity and MoA [62]. These assays have proven to be a viable assay option for detecting NAbs against a wide variety of biotherapeutics [63, 64]. Alternatively, non-cell-based assays (e.g., competitive ligand binding assays with recombinant target, such as soluble target or receptor-Fc fusion proteins) are highly reproducible and relatively easy for a trained analyst to perform and validate [50]. This approach is recommended when evaluating the ability of antibodies to inhibit ERT drug enzyme activity, as in FD [65], since the assays measure enzyme capacity to metabolize substrates and help determine drug concentration needed to counteract inhibitory antibodies [65]. However, when measuring IgG in FD, it is still important to perform cell-based assays to determine whether the antibody can interfere with M6P receptor binding and with intracellular uptake [33].

Myler et al. (2023) outlined key validation criteria for NAb assays [50]:

- Inclusion of relevant controls in the assays (low positive control (LPC), high positive control (HPC), blank control, drug control, and ligand control,)
- Ccoefficient of variation (%CV) between replicate wells ≤ 25 %. [54]
- Setting statistical limits based on pre-study validation (Rank between the HPC, LPC, and the cut point [CP]).

To establish Upper and/or lower limits the following equation are applied:

- Upper limit: mean + t(0.01, n-1) × DE.
- Lower limit: mean − t(0.01, n-1) × DE.

In these equations, the mean and standard deviation (SD) are calculated using the data from all control samples tested during pre-study validation; t(0.01, n-1) is the critical value from the one-sided t-distribution with *n-1* degrees of freedom, corresponding to a 1% error rate, and *n* represents the number of independent replicate results used in this evaluation.

These and other recommendation are included in the guidelines provided by the FDA and other regulatory agencies for assay validation [17, 54].

### Clinical impact

There is a demonstrated deleterious impact of NAbs on the long-term efficacy of treatment in classic FD patients [38, 66, 67]. The formation of ADAs results in attenuated efficacy [21] by inhibition of α-Gal A activity and enzyme uptake and processing by cells. The added negative impact on the PKs of the infused enzyme, limits its biocellular dynamics and response [20, 27, 32, 60, 68]. Plasma lyso-Gb3 and other biomarkers show a negative trend [40]. Monitoring the presence of NAbs in FD patients under ERT should be mandatory [38, 39].

ADAs monitoring should focus on understanding the potential correlation between ADA response and its impact on clinical safety and available mitigation strategies [55].

ADAs generation has been identified as one of the main causes for discontinuation of protein-based therapies due to therapeutic failure and/or side effects [69, 70] Overall, reports of increased ADAs titers and consequent reductions in enzyme activity or plasma lyso-Gb3 clearance have been described and demonstrated [38, 55] with both agalsidase alfa and agalsidase beta [20, 28, 41, 60, 71–76]. Although a lyso-Gb3 reduction in plasma does not guarantee a clinical response in all the FD patients and all the affected organs, the absence of a lyso-Gb3 response upon treatment iniciation may suggest a loss of therapeutic efficacy [20]. In a slow and chronic disease like FD, the clinical outcome under therapy is very difficult to evaluate in a short term. In addition, it is influenced both, by NAbs and, on the other hand, by the severity of the visceral damage before therapy initiation [27] In the future, to better differentiate the NAbs impact, the study populations being compared should have a similar phenotype disease burden before therapy initiation and comparable types of genetic variants.

The appearance of ADAs in ERT treated patients has also been associated with higher amount of Gb3 deposits in endothelial cells of the skin [77]. Different case studies have confirmed the deleterious effects of ADAs by showing massive Gb3 storage in various tissues in an affected male patient even after 6 years of ERT [78], and increase of lyso-Gb3 levels and lack of improvement in symptoms such as abdominal pain and acroparesthesias were evident in two men after 3 years of ERT with high antibody titers [79].

Recent studies have shown that ADAs positivity to significant titers were associated to worse renal function [38, 55], higher left ventricular mass, worse disease severity scores and deterioration of quality of life [55].

ADAs positivity against agalsidasealfa or agalsidasebeta in male classic FD patients and non- saturation of therapeutic ADAs, was associated with a marked decrease in eGFR compared with those with ADAs saturation [55]. In a study of 26 males with classic FD receiving ERT for a median of 94 months, a non-saturated ADAs status during infusion was associated with progressive decrease in eGFR and ongoing cardiac hypertrophy [80]. Currently, plant-derived ERTs have been developed to reduce ADAs development and improve enzyme biodistribution [81]. Pegunigalsidasealfa, a novel pegylated formulations of α-GAL, has a longer circulatory half-life and increased heart and kidney uptake compared with current ERTs [32, 81]. It has also been suggested that the unique PKs profile and the apparent attenuated immune response demonstrated by pegunigalsidase alfa may improve safety and clinical response in FD patients [32]. This is supported by the favorable adverse effect profile, stability of renal function and cardiac parameters, and improved disease symptoms observed in participants in Schiffmann’s study during the 12-month treatment period [32].

Additional evidence in real life and consensus are required to develop strategies related to the treatment switch and the potential use of immunomodulatory therapies [20].

### Impact on safety

The ERT efficacy and safety depend, among other causes, on the titers of ADAs in circulation, their neutralizing ability and immunochemical properties of drug-ADA ICs, both in plasma and in tissues [82]. The most common ERT-related adverse events are IRRs, which are generally mild or moderate, and tolerable in most cases [23, 44, 83, 84], basically limited to fever and shivers [37], headache, paresthesia, decrease blood pressure, nauseas, and fatigue may also appear—particularly after infusions [29]. These symptoms can be easily managed with low doses of nonsteroidal antiinflammatory drugs, antihistamines and/or glucocorticoids administered before the infusion, as well as decreasing the infusion rate [28, 29]. IRRs rarely require ERT discontinuation, they subside over time, and seldom interfere with ongoing ERT [24, 84, 85]. Anaphylaxis is rare, although potentially life-theatening reactions have been reported [24].

IRRs occur in 14% of patients treated with agalsidase alfa [14]; in 67% of patients treated with agalsidase beta [15], and in 21% of patients treated with pegunigalsidase alfa [16], according to the summary characteristics of each product. However, the studies and patient populations reviewed may not be comparable.

The risk of IRR seems higher in men with absence or very low enzymatic activity and null or nonsense mutations [37]. According to Nicholls and collegues [24] IRRs are less common in patients with missense mutations, in whom low levels of endogenous enzyme can usually be detected. Regarding the appearance of ADAs, men who develop anti-αGAL A immunoglobulin G (IgG) antibodies are more likely to experience IRRs than those who remain seronegative [26, 37, 38], as shown in an analysis of 571 men from the Fabry registry [26].

Recent results have been published on IRRs in patients treated with pegunigalsidase alfa. In the study by Mehta and colleagues [86]. (n = 111) IRRs were observed in about one-fourth of patients who had not previously received ERT or had switched to ERT after treatment with pegunigalsidase alfa. Most events were mild to moderate and occurred during or withing 2 h of the drug administration. Despite IRR rates being generally low, they roughly doubled in patients who had never received ERT, consistent with observations where IRRs mainly occur during the first year of ERT [85]. In another study (Hughes and colleagues) [87] where IRR occurrence was assessed in 141 patients treated with different doses of pegunigalsidase alfa (1 mg/kg each other week or 2 mg/kg each 4 weeks), they predominantly appeared within the first year of the treatment initiation and affected fewer than one-third of treated patients. Most IRRs occurred during infusion and up to 2 h later in patients with pre-existing or de novo ADAs, in both dosing groups, and the frequency decreased over time parallel to the decrease in premedication use among those patients who had switched ERT. The higher IRR rate observed with the dose of 2 mg/kg every 4 weeks versus the dose of 1 mg/kg every other week was largely associated with male patients who had baseline ADAs with a longer previous exposure to ERT. Although ADAs pre-existence from previous ERTs posed a higher risk of IRR, these patients experienced IRRs less frequently after the first year of treatment with doses of 2 mg/kg every 4 weeks.

IgE-mediated reactions are very rare in patients under treatment with ERT. They have been described during treatment with several ERTs [4, 15, 47]. Some studies suggest that these reactions appear with some ERTs but not others [88]. A standardized review of all ERTs [89] is necessary, since the summary of product characteristics of all three ERT on the market warns of the possibility of hypersensitivity reactions [14–16].

IgE-dependent immune responses are not the only ones causing most IRRs in FD [90]. Anaphylactoid rather than anaphylactic reactions is a real issue [20]. The mechanisms and underlying immune perturbations resulting in hypersensitivity to the infused enzyme are not yet fully understood [90]. The study by Limgala and colleagues in 8 patients who experienced an IRR during treatment with agalsidase beta suggests an interference between immune cells and complement factors. IgE-independent specific degranulation of mast cells [90] and an abnormal activation of the complement system can result in a type II hypersensitivity reaction [27]. In this study, only 25% of patients with IRRs had reduced C4 levels, suggesting that the primary cause for IRRs is not complement-mediated cytotoxic hypersensitivity reactions [90]. According to the authors, mast cell stabilizers may be used to control IRRs and to safely reintroduce agalsidase in patients previously treated with ERT [90].

To conclude, it must be highlighted that in patients experiencing severe IRRs and requiring potent premedication including high doses of corticosteroids prior to infusions this may have an impact on ADAs formation, as previously has been demonstrated in patients undergoing immunessupression for kidney and heart transplantation [80].

### ADAs interference mechanisms

NAbs are a subset of ADAs that inhibit binding of the biotherapeutic to its target. This inhibition may result in the neutralization of the biotherapeutic’s pharmacologic activity, potentially reducing efficacy. For biotherapeutics that are homologous to endogenous proteins with non-redundant function NAbs may inhibit ERT efficacy in two ways: through the inhibition of the ERT uptake into the targeted cell or by inhibiting the ERT catalytic activity [91]. The clinical repercussions of Ab formation against biologicals depend on the ADA epitope. NAbs bind to the active site of the drug, inhibiting its MoA. Non-neutralizing Abs, however, bind to the drug without neutralizing it. In both cases, the kinetics of the drug are modified, as the formation of drug-ADA ICs accelerates its elimination, affecting therapeutic efficacy [92].

Several studies have shown that inhibitory ADAs developed against infused α-Gal A belong to IgG1 and IgG4 subclasses [41, 55, 66]. Depending on the recognized epitopes, ADAs seem to have an impact on several different mechanisms [27]. In general, the uptake of infused agalsidasealfa or beta is mannose-6-phosphate (M6P) receptor-mediated, resulting in increased lysosomal α-Gal A activity and subsequent Gb3 depletion. In addition, infused α-Gal A can directly deplete Gb3 and lyso-Gb3 directly within the plasma [27]. In the presence of NAbs, infused α-Gal A is recognized by ADAs, resulting in different effects. ADAs may recognize epitopes localized at uptake-relevant domains including amino acid positions N139, N192, and N215 [27]. The binding of ADAs may result in a masking of these positions, which are subsequently not accessible to the M6P receptor, resulting in a decreased enzymatic uptake followed by a reduced Gb3 depletion [60]. To date, the most well-characterized function of ADAs is their ability to neutralize enzymatic activity during infusion [39, 40, 66, 67], probably mediated by the recognition of epitopes within catalytic domains. First, activity-neutralized α-Gal A will not be able to reduce Gb3 and lyso-Gb3 in the plasma. Second, even if these inactive ADA/α-Gal A complexes are internalized and translocated to the lysosomes, they seem to be unable to dissociate [60]. BAbs may also affect PKs, trafficking, and conformation, which is also currently yet insufficiently analyzed in FD [27]. Finally, Fcγ-receptor-expressing cells, such as macrophages, recognize drug/ADA complexes via the Fc- fragment, and lead to increased drug clearance [39].

#### Role of immune complexes

Although not directly demonstrated in FD, ADAs might also form large ADA-protein ICs, which are insoluble and can mediate complement activation and result in membranous nephritis as reported for Pompe disease [93–95]. Since ADAs in affected FD patients are polyclonal and thus recognize different epitopes [60, 96], it can be assumed that in most individuals a combination of the mechanisms leads to decreased therapeutic efficacy [27].

Many of the immune-mediated adverse effects attributed to ADAs require the formation of a drug/ADA IC intermediate, which can have a variety of downstream effects [97]. Therapeutic molecules bound to cell surface protein targets may also attract circulating ADAs leading to formation of ICs on cell membranes in tissues [97]. ADAs bound to drug in circulation gives rise to circulating ICs (CICs) which can result in type III reactions. Regardless of their presence, ICs are relevant from two main points of analysis their size and their propensity to activate complement. Both factors can drive formation of IC deposits and activation of inflammatory pathways [97].

In summary, ADAs may reduce the drug efficacy by competing with the endogenous ligand and/or forming an IC, which accelerates the drug clearance from circulation and reduces its bioavailability, and so is a factor that should be considered, especially in the case of long-term treatment and when assessing secondary loss of response [35].

### Risk factors to develop ADAs

There are several patient- and product-specific factors that need to be considered in the assessment of the immunogenic risk for FD patients treated with ERT [27].

#### Drug-related factors

*Administration route*

Subcutaneous administration allows for extended contact of the molecule with dendritic cells, and so it is more immunogenic than endovenous administration [98]. In FD, the three currently approved products agalsidasealfa, agalsidasebeta and pegunigalsidasealfa are intravenously infused and are associated with the lowest risk of triggering an immune response [17, 23, 32, 83].

*Dosage, frequency of administration and duration of exposure*

The dosage of ERT also plays a relevant role, since it is more likely for higher doses to increase the risk of generating ADAs [97, 99]. The frequency and duration, repeated dosages, and prolonged exposure may either break or lead to tolerance [97, 99]. Short-term treatment is usually less likely to be associated with a harmful immune response than a long-term treatment [17]. Re-exposure after a long treatment-free interval may be associated with an enhanced immune response [17].

Additionally, as demonstrated with agalsidase beta, the patient seroconversion is very limited by maintaining a low infusion rate in the first months, when the probability of onset of the development of antibodies is higher [100].

*Manufacturing process, formulation, and stability characteristics*

Product-specific factors may include host cell protein contaminants and non-human post-translational and chemical modifications of the protein [17, 97, 99], as well as the presence of impurities [17]. The immunogenicity risk of host cell proteins is dependent on the source (cell line) of the therapeutic protein [17].

In addition, "artificial" modifications of medical products, such as glycosylation, amino acid substitutions, or pegylation, may impact the immune response. Ultimately, immunogenicity might be influenced by a combination of factors; for example, the higher appearance of ADAs in patients treated with agalsidase beta versus agalsidase alfa may be attributed to the higher dose (1 mg/kg versus 0.2 mg/kg), as well as the difference in the production cell line, and therefore, the glycosylation pattern, or a combination of both [68].

#### Patient-related factors

*Genetic factors*

When the therapeutic protein is used to substitute of an endogenous protein in patients who are deficient, partially deficient, or carriers of a modified form of the natural counterpart, the physiological antigen may represent a neo-antigen, and the immune system will interpret the therapeutic protein as foreign or non-self [17]. One of the most important factors is the CRIM status, defined by the presence or absence of endogenous α-Gal A. CRIM-negative patients without endogenous enzyme have the highest risk of producing antibodies under therapy [27]. Since FD is an X-chromosomal-linked disease, only hemizygous males or homozygous females (very rare) can be CRIM negative [27]. Therefore, classical male FD patients with nonsense or null mutations, which result in the absence of any detectable α-Gal A (functional or non- functional) are at a high risk of forming ADAs [27, 39, 67].

*Pre-existing immunity*

Pre-existing antibodies (Pre-Abs) are endogenous antibodies that are either specific to or cross-reactive with epitopes on proteins or glycans that overlap with therapeutic protein epitopes. Pre-Abs may result from previous exposure to similar or related proteins but can also be found in treatment-naïve patients [17].

A distinction must be made in FD between anti-α-Gal A antibodies, which are produced against the enzyme’s peptide chain, and antibodies against specific fractions that may be bound to that enzyme, such as polyethylene glycol (PEG) residues present in pegunigalsidase. In both cases, previous exposure to enzyme (e.g., in patients who previously received another ERT) or to PEG (present in many drugs, as well as hygiene and cosmetic products) may result in a patient presenting with pre-existing antibodies before exposure to the new enzyme.

In pegunigalsidase alfa clinical trials, 25% of patients presented pre-existing ADAs before receiving the first dose of the drug due to previous exposure to other ERTs [48]. Additionally, antibodies generated specifically against the polyethylene-glycol part (PEG) of pegylated proteins have been identified, including pre-existing anti-PEG antibodies [17]. The frequency of pre-existing anti-PEG antibodies in FD patients is comparable to that observed in the general population. Due to the low titers, these pre-existing antibodies had minimal effect on the pegunigalsidase alfa activity *in vitro* [66], and a recent study observed they had no significant impact on the half-life [66]. Furthermore, a tolerability analysis of pegunigalsidase alfa treatment in FD patients vaccinated against COVID-19 suggests that there is no higher risk of IRR after receiving pegylated vaccines (e.g. tozinameran, Pfizer-BioNTech; mRNA-1273, Moderna), since no IRR were reported following vaccination with any of these vaccines, including pegylated ones, and no anti-PEG antibodies were identified after the administration of pegylated vaccines in patients treated with pegunigalsidase alfa. PK parameters of pegunigalsidase alfa were not altered either after the vaccines [101].

*Other patient-related factors*

*Immune status and immunomodulatory therapy:* Much of the variability in the propensity of administrated treatments to induce ADAs formation may result from different immune contexts [36]. Principally, disease status and human leukocyte antigen (HLA) alleles, which could promote or inhibit an ADAs response. The idea that ADAs formation is often derived from a T-dependent response has recently led to studies focusing on how ADAs formation correlates with HLA polymorphism in the population [36].

*Age.* Data on immunogenicity from one age group cannot necessarily be projected to others, since immune response to therapeutic proteins can be affected by patient age. Among the pediatric population, different levels in the immune system maturation are seen depending on age, and discrepant immune responses to a biological product may be expected [17].

*Concomitant treatments*. Concomitant therapies may either decrease or increase the risk of an immune response to a therapeutic protein. Typically, the immune reaction against a therapeutic protein is reduced when immunosuppressive agents are used concomitantly. [17]

### ADA cross-reactivity among different ERTs

Two ERT preparations have been marketed in Europe for over 20 years: agalsidase alfa (Replagal®, Takeda) and agalsidase beta (Fabrazyme®, Sanofi) for the treatment of FD [102]. More recently, the use of a new ERT, pegunigalsidase alfa (Elfabrio®, Chiesi) [16], was approved.

#### Cross-reactivity between agalsidase alfa and agalsidase beta

Although these formulations are biochemically and structurally very similar [103], agalsidase beta contains oligosaccharides with a higher content of sialic acid and more M6P than agalsidase alfa. Because the entry of recombinant agalsidase into cells is mediated by M6P receptors, greater absorption of agalsidase beta than alfa has been showed both in vitro and *in vivo* [102]. Moreover, they are produced using different methods and are approved at different dosages [104]. Even though agalsidase alfa and beta have epitopes in common, which may suggest a similar immunogenic profile, agalsidase alfa is produced using cultured human skin fibroblasts, whereas agalsidase beta is produced by the expression of human α-galactosidase A cDNA in Chinese hamster ovary cells [105]. Despite their similar amino acid composition and biochemical properties, different patterns of posttranslational glycosylation of mannose residues have been noted, which alters the uptake of these preparations into tissues and could lead to differences in antigenicity [106]. Both preparations are safe and effective in patients with FD, but very few data directly compare the clinical effects of the two drugs [102].

The worldwide shortage of agalsidase beta supply (from June 2009 to January 2012) because of a viral contamination in the manufacturing process of Fabrazyme® resulted in a switch of therapy from agalsidase beta (1.0 mg/kg) to agalsidase alfa (0.2 mg/kg) [107–109] offering the possibility to compare, although indirectly, the effects of the two drugs [102].

Four studies, with a total of 51 patients, assessed the potential appearance of ADAs after switching from agalsidase beta to agalsidase alfa [106, 110–112]. Tsuboi and colleagues [106] reported just one allergic reaction related to the agalsidase beta treatment in a patient who tested positive for antibodies against agalsidase beta. This reaction was not observed after switching to alfa. Conversely, all patients tested negative for anti-agalsidase alfa antibodies before the switch, including the patient with anti-agalsidase beta antibodies, who remained negative after 36 months [106].

Skrunes et al [113] reported that virtually no patient (n = 20) developed neutralizing antibodies when switching agalsidases (alfa to beta or vice-versa). Only one patient became positive for NAbs after 10 years of agalsidase alfa treatment and reported a transient doubling of the titer after switching to agalsidase beta.

The INFORM study assessed the potential appearance of cross-reactivity when switching treatment with agalsidase alfa to agalsidase beta in 15 patients [112]. Prior to the first agalsidase beta infusion, eight patients were positive for serum IgG antibodies to both agalsidase alfa and agalsidase beta when the same assay format was used, although some patients had never been treated with agalsidase beta. Seven patients were seronegative for IgG antibodies to both agalsidase beta and agalsidase alfa, four of whom had been treated with both agalsidase alfa and agalsidase beta. No patient was positive for antibodies against one ERT while remaining negative for the other. Titers of antibodies to agalsidase alfa and agalsidase beta at baseline were not significantly different, even in the six patients who had not yet been exposed to agalsidase beta. Only one seronegative patient seroconverted during the agalsidase beta study treatment period. In patients who were positive for agalsidase beta antibodies, there was no discernible pattern between the titer measured at any time point during the study and concentrations of lyso-Gb3, plasma Gb3, or urine Gb3. Patients who were negative for antibodies at baseline were among those with the lowest plasma concentrations of lyso-Gb3 and Gb3 [112]. Similarly, in another group with 15 patients who switched from agalsidase alfa to beta, Limgala et al [111] reported that 6 patients tested positive for anti-agalsidasa alfa ADAs during both ERT periods with 3 of these patients also testing positive for neutralizing antibodies. No other patient seroconverted after switching.

It must be noted that most studies did not observe any change in plasma lyso-Gb3 levels upon switching from agalsidase beta to agalsidase alfa [106, 112, 114, 115]. Additionally, the study by Lenders and colleagues [115] showed a significant decrease of lyso-Gb3 levels upon switching back to agalsidase beta after a first switch from beta to alfa. To exclude a solely neutralizing effect of ADAs on the α-Gal A enzyme that may affect the lyso-Gb3 level after a re-switch, the authors further analyzed a subset of male patients with available lyso-Gb3 data and ADAs. It must be noted that, in this study, the lyso-Gb3 decrease was more prominent in patients with NAbs [115].

Both agalsidase alfa and agalsidase beta induce similar, fully cross-reactive, antibody responses *in vivo* [39, 103, 116], refuting the suggestion that variation in glycosylation patterns between agalsidase beta and agalsidase alfa may have implications for the long-term safety of ERT [117]. The perceived differences in immunogenicity of the two enzymes could be based on differences in analytical testing methods [112].

#### Cross-reactivity between agalsidase alfa or beta and pegunigalsidase alfa

In a recent study by Lenders and colleagues [33], cross-reactivity of ADAs against agalsidase alfa and agalsidase beta was analyzed in 49 FD patients, against the new enzyme pegunigalsidase alfa. The main findings were that, first, pre-existing ADAs against agalsidase alfa and beta showed significantly lower affinity for pegunigalsidase alfa; secondly, the lower affinity also resulted in a reduced inhibitory capacity (30% less) of pre-existing ADAs against pegunigalsidase alfa and less impact on cellular uptake by pre-existing ADAs against pegunigalsidase alfa; and last, this reduced affinity could be explained by masked epitopes due to pegylation rather than agalsidase alfa- or beta-specific epitopes of pre-existing ADAs. In short, the study demonstrates lower affinity and enzyme inhibition (included cell activity and uptake) of pre-existing ADAs against pegunigalsidase alfa, which results in a decrease of ADA-mediated enzyme inhibition and a reduction of ADA interference with the enzyme uptake. Therefore, treatment with pegunigalsidase alfa could be a promising therapeutic option for most patients with pre-existing ADAs, but patients should be tested before the switch in order to better predict the therapeutic success [33].

### Strategies to minimize ADAs development

. To minimize the development of ADAs, two hypothetical approaches would be possible: through ITI in ADA-positive patients, or by preventing ADAs formation prior to treatment [68]. Both strategies have previously been tried in other diseases [68].

#### Immune tolerance induction (ITI)

Lenders et al. treated with immunosuppressive therapy FD patients who underwent kidney or heart transplantation. In this study, patients with established ADAs demonstrated an initial reduction in their ADAs titer. However, after tapering of the immunosuppressive medication (specifically corticosteroids), ADAs titers increased again [80]. Therefore, patients would require continuous exposure to immunosuppressive drugs to maintain immune tolerance, with unacceptable side effects for a slowly progressive disease like FD [68]. In the study by Lenders and colleagues (2017), six male patients with classic FD who started ERT after a transplant, did not develop ADAs [80].

Additionaly, a recent study demonstrated tolerance induction of agalsidase beta in eight patients (only one IgE-positive and six patients with IgG against α-Gal A) using a combination of premedication that included corticosteroids, mast cell stabilizers, H1 and H2 blockers and intravenous fluids [90].

Aydin and colleagues [118] used an adapted three-bag protocol [119] which was based on a consecutive 1:10 dilution of 14 mg agalsidase alfa solved in 250 mL normal saline solution to successfully reinstitute agalsidase alfa infusion in two brothers negative for IgEs [118]. This protocol starts with 1/40,000 of the therapeutic dose and increases dose over 12 steps, resulting in an infusion time of approximately 504 min [118]. However, a recent case study demonstrated that a three-bag protocol alone or in combination with pretreatment including glucocorticoids, H1/ H2 antagonists, leukotriene receptor antagonists, and even B-cell depletion via rituximab was not able to reinstitute agalsidasebeta in a patient with IgEs against α-Gal A but required a specific anti-IgE inhibition by omalizumab [120].

Some studies have demonstrated that ADAs titers can saturate during infusions [67]. As an alternative approach, patients with moderate ADAs titers might benefit from an approved dose escalation (i.e. switch from agalsidase alfa [0.2 mg/kg body weight] to agalsidase beta [1.0 mg/kg body weight]) to saturate all free ADAs. It is still unclear whether dose escalation could also trigger further antibody production in the long term. However, some studies demonstrate that even in patients with ADAs a higher dose of infused enzyme has a beneficial effect on the decrease of lyso-Gb3 concentration, as well as disease progression [66, 67]. A switch from agalsidase alfa to agalsidase beta appears to have heterogeneous long-term effects in patients and seems to trigger an increase in ADAs titers after the change but could lead to durable ADAs saturation (in more than a quarter of patients) [121].

#### Prevention of ADAs formation

FD patients with NAbs might benefit from immune modulatory protocols to reduce titers [80], but aggressive therapeutic strategies as applied for other lysosomal diseases [57, 122] would be accompanied by (severe) side effects and are therefore not yet implemented in routine clinical practice for FD patients.

Another approach to ADAs prevention is treatment initiation with lowerand more regulardoses of recombinant protein. A study by Van der Veen and colleagues (2020) [68] demonstrates that treatment with lower doses of agalsidase alfa is associated with a lower risk of ADAs development. Thus, starting treatment with lower than registered doses of recombinant enzyme, in combination with shortened administration intervals, might be a way to induce central tolerance. Once central tolerance is induced, doses could gradually be increased and dosing intervals reduced. [68]. In patients with IgE against agalsidase beta, a gradual dosage and individualized infusion rate regime during 27 infusions resulted in the successful reinstitution of agalsidase beta in five out of six patients [22].

Last, a recent study (Mignani y cols. 2024) [100] assessed the effects of a new agalsidase beta infusion protocol in 31 FD patients, considering not only the safety but also the immunogenic and clinical consequences of reducing infusion time. With this aim, patients were split into two phases. In the first phase (stable phase), the infusion rate was 15 mg/h for the initial 8 infusions, as the early months of agalsidase beta infusion would be the most critical for the development of ADAs [26, 39, 40]. Afterward, in the subsequent 8 treatments, the infusion rate was progressively increased (escalation phase), setting a limit of infusion rate not higher than 0.8 mg/min (48 mg/h), which is equivalent to an infusion time of two hours for a standard treatment (patient of 70 kg). The study included both treatment-naïve patients and patients previously treated with agalsidase alfa (seronegative at baseline) as well as patients with classic or late-onset phenotypes of both genders. Only one episode of mild IRR was observed, and a cumulative incidence of ADA-positive patients of 19.4% (6 patients). Four of these patients presented a classic and two a late-onset phenotype, and in 50% of them, the antibodies disappeared 1 year after starting agalsidase beta. This study would seem to demonstrate that reducing the agalsidase beta infusion time is possible and safe, either from an immunogenic or a clinical standpoint. The reduction of agalsidase beta infusion time, compared to the Summary of Product Characteristics [15], does not seem to increase the patient immunogenicity, as shown by the low incidence of ADAs generation despite a higher infusion rate [100]. However, some limitations to this study must be considered, such as the absence of a control group of patients treated with the infusion rate approved in the Summary of Product Characteristics, the relatively small sample size, and the limited follow up duration (1 year) [100].

#### Future strategies

Currently, in vitro studies are being conducted through directed evolution techniques to generate more stable α-Gal A variants as potential next generation treatments for FD [123]. This technique emulates the natural evolution process, but at lab scale: through successive cycles of random mutagenesis, DNA shuffling and selection, more powerful and versatile enzymes are achieved.

Hallows and colleagues (2023), in addition to introducing mutations that improve stability, identified and incorporated mutations that reduced or eliminated in silico-predicted immunogenic epitopes, thus confirming a reduction in putative epitopes by MHC-II (MHC-associated peptide proteomics, MAPP) [124].

### Limitations and barriers in ADAs detection

Before any further consideration of ADAs and their role in FD patients and ERT, current limitations should be acknowledged to determine them in an accessible and efficient way in clinical routine [35]. One of the problems in determining ADAs is the concomitant presence of the drug in the serum [125]Hence, determination must always be carried out when drug concentrations are presumably at their lowest— i.e., at the time prior to administration— to avoid interference with the results.

Despite the progress of bioanalytical methods, subsequent steps in the investigation of immunogenicity can be invalidated by suboptimal immunogenicity assays [126]. The development of valid assays is critical as it is the foundation upon which all subsequent assessments are built [126]. To develop assays for studying the inhibition of infused protein, a comprehensive understanding of the nature of protein, its MoA and its target (cellular and molecular) is required [122]. The assays should be quantifiable and clinically relevant, mirroring the in vivo interaction of the infused protein and antibodies [122, 126].

The determination of ADAs is not easily comparable across different laboratories. The reasons making this comparison so difficult are multifactorial. In ELISA-based assays for antibody measurement, the absence of a reference antibody necessitates expressing results as relative titers, using serial dilutions of samples compared to a negative control. Commercially available antibodies could be used for absolute quantification; however, they recognize only a limited set of epitopes that may not fully represent the ADAs in the samples. Additionally, these antibodies are typically produced in non-human species, requiring a different detection system than the one used for patient samples [49].

A demonstration of the practical difficulty in determining ADAs and comparing the results obtained is the existence of different ELISA tests available. Although ELISA tests are currently the gold standard for ADAs detection [127], there are different types that, to a greater or lesser extent, are subject to limitations. In a direct ELISA, ADAs from patient samples are captured by the biopharmaceutical, which has been immobilized on a plastic plate; the plate is washed several times and ADAs are then detected spectrophotometrically using a colorimetric labeled anti-immunoglobulin reagent. A limitation of the direct ELISA is that the fixation of the biopharmaceutical to a plastic surface may alter its conformation and conceal epitopes, resulting in underestimation of ADAs. The indirect ELISA format circumvents this complication by first immobilizing antibodies on the plate to orient the biopharmaceutical. Disadvantages of the direct and indirect ELISA formats include false positives and high background noise due to non-specific binding as well as potential loss of low-affinity ADAs during washes. In a capture ELISA, ADAs are captured by immobilized biopharmaceutical and then detected using a conjugated version of the biopharmaceutical. This version is more selective and specific than the direct or indirect formats, but the possibility of losing low-affinity ADAs remains [128, 129].

As discussed in other sections, the impact of ADAs on clinical efficacy is to diminish the activity of the drug, either by blocking (neutralization) or enhancing clearance [130]. Substantial amounts of ADAs will be required to inactivate a major part of the drug, which is usually administered in high doses, thus the impact of ADAs on clinical efficacy critically depends on the amount of ADAs relative to the amount of drug [130]. Therefore, another limitation in clinical practice is that merely detecting ADAs will, in general, not directly relate to possible clinical implications, and so the prediction of loss of response or adverse events due to ADAs remains challenging. [130].

Considering the above, the detection NAbs is the most important aspect in the assessment of immunogenicity [131], as NAbs determination is a functional assay that assesses the inhibition of therapeutic activity [53]. However, developing and validating NAbs assays showing a direct interference with a biological function remains a challenge [131]. Existing limitations may complicate data interpretation and lead to false negatives, due to methodological differences [131].

The ADAs assay sensitivity and drug immune tolerance strongly depend on the affinity/binding characteristics of the selected positive control (antibody) and are not fully representative of the broad variety of ADAs in study samples [126]. This is why the development of assays with high sensitivity, precision, and specificity for ADAs has become critical for the quality control of biopharmaceuticals and in examining the treatment of patients with these drugs [132]. Furthermore, developing a relevant integrated analysis strategy for the treatment plan is fundamental to elucidate the clinical relevance of immunogenicity data [17]. The controversial results of ADAs on treatment efficacy may be explained by the fact that no standardized assays and protocols have yet been developed to determine IgG levels in affected FD patients [27, 133]. Thus, as discussed, titers from different studies are hardly comparable [27]. Another pitfall in many studies is the lack of a corresponding methods section describing the protocols used.

The results of ADAs detection assays facilitate the understanding of immunogenicity, PKs, PDs, safety, and efficacy of therapeutic protein products. Although information on ADAs incidence is typically included in the prescribing information of each product, the FDA cautions that comparing ADAs incidence across products, even for products that share sequence or structural homology, can be misleading because detection of ADAs formation is highly dependent on the sensitivity, specificity, and drug tolerance level of the assay [54]. Therefore, comparing immunogenicity rates across therapeutic protein products with structural homology for the same indication is unrealiable, even though fully validated assays are employed. When a direct comparison of immunogenicity across different therapeutic protein products that have homology—or across similar therapeutic proteins from different sources—is needed, the comparison data should be obtained by conducting a head-to-head clinical study from which samples obtained are tested using an assay demonstrated to have equivalent sensitivity and specificity for antibodies against both therapeutic protein products [54].

Regulators have promoted high-sensitivity assays to assess the immunological tolerance of medications, but this has led to some interpretation challenges. For instance, assays with higher sensitivity detect more ADAs, but these may not correlate with the loss of efficacy and/or clinical impact if the analysis is carried out simply by comparing ADA-positive and negative populations [126]. In these more sensitive assays, there is a risk of losing correlations between ADAs positivity and the clinical impact.

Furthermore, the presence of ADAs may sterically hinder detection of drug in the PK assay and therefore it may seem that there is an impact on exposure when there is not. However, it should also be noted that if ADAs affects the drug’s detection in the PK assay, they likely also affect its ability to bind to the target. Characteristics of ADAs responses (e.g., persistent vs transient responses, titer levels, etc.) may be more relevant to assess correlations with efficacy, safety or PKs than ADAs incidence alone [126].

Nevertherless, despite the numerous limitations expressed, the presence of NAbs could still impact clinical outcomes, regardless of the general assay used for the measurements. The correct interpretation of the impact of ADAs on therapy efficiency and clinical outcome in patients with FD should probably only be performed when analyzed patients are stratified according to supersaturated ADAs titers during infusion [134].

## International recommendations on ADAs monitoring in FD patients

According to the document *Guideline on Immunogenicity assessment of therapeutic proteins*, published in 2017 by the EMA [17], potential immunogenicity to the treatment with a therapeutic protein should be systematically tested in patients by scheduled routine repetitive sampling. Additionally, immunogenicity should be determined in a symptom-driven manner, for instance, when an unwanted immune response is suspected [17]. The frequency of sampling as well as the timing and extent of analyses will also depend on the risk assessment for a particular treatment [17].

Sampling schedules should be designed to distinguish patients being transiently positive from patients developing a persistent antibody response. Post-treatment follow-up sampling should be long enough to allow conclusions on the persistence of the immune response triggered by the therapeutic protein and to uncover any immune reaction that may have been suppressed by the therapeutic protein itself. The timing of post-treatment sample(s) is determined by the half-life of the protein and the drug tolerance of the ADAs assay [17].

More frequent sampling is necessary in the earlier phase of treatment, where patients are normally most at risk of ADAs development. However, it is interesting to consider long-term follow-up of immunogenicity through less frequent ADAs determinations, as this strategy provides additional information on the evolution and consequences of immunogenicity [17].

**Fig. 1:**
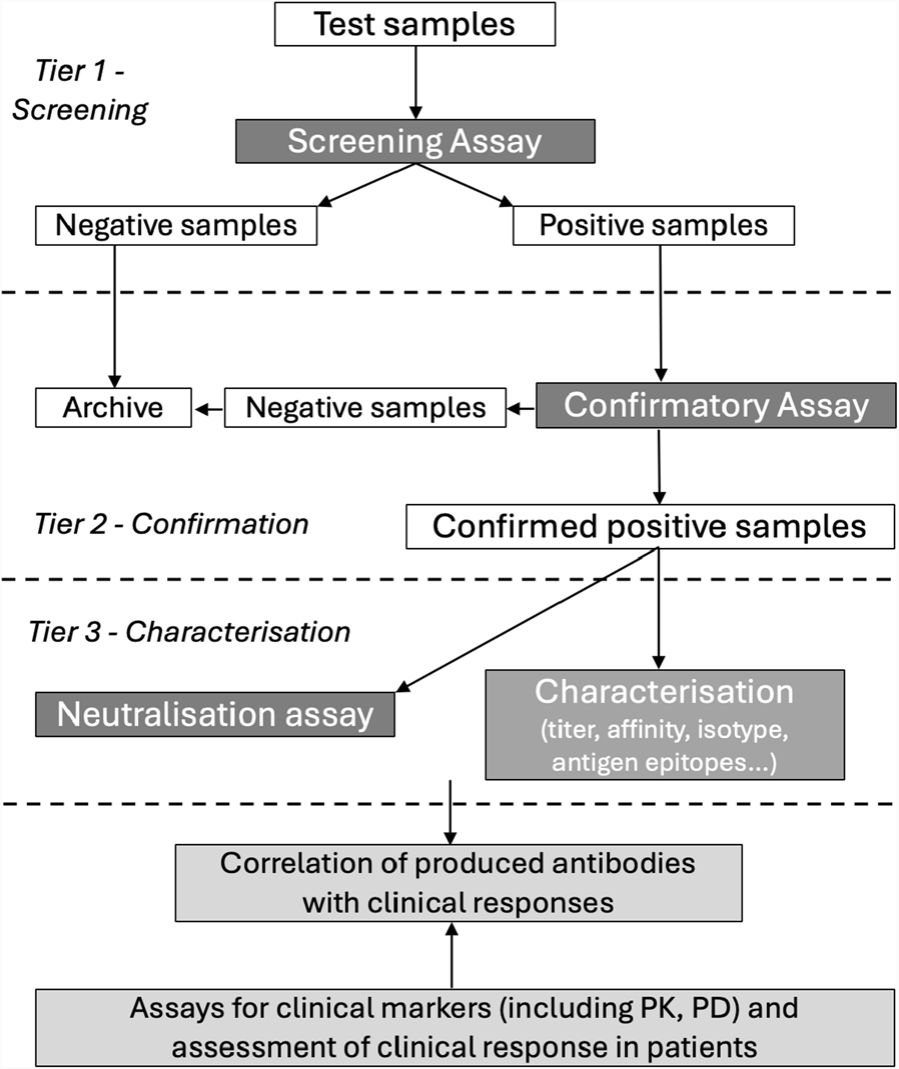
An example of a strategy to assess the immunogenicity of a therapeutic protein in clinical practice *(modified of Committee for Medicinal Products for Human Use (CHMP), “Guideline on Immunogenicity assessment of therapeutic proteins,” 2017)* [17]. The algorithm establishes a multi-tiered approach. In a first phase, a screening assay should be conducted to identify ADA-positive samples/patients; in a second phase, a procedure for confirming the presence of ADAs and determining their specificity, and last, a third phase with functional assays for the assessment of the neutralizing capacity of antibodies. Screening assays are, therefore, the first step in the assessment of immunogenicity. These assays should be sensitive and capable of detecting all clinically relevant antibodies (including IgM and IgG subclasses) induced against the product in all ADA-positive patients [17]

According to the document mentioned, immunogenicity to therapeutic proteins should be assessed using assays validates according to the scheme below (Fig. 1):

Samples taken 7 to 14 days after the first exposure can help elucidate an early IgM response. Samples taken at 3 to 6 weeks after the first exposure are generally optimal for determining IgG responses [54]. It must be noted, however, that the ideal time to assess the potential existence of ADAs is right before the administration of the next dose, when the drug levels are presumably at their lowest [35].

Recently two expert consensus guidelines have been published on FD [20, 21]. In both cases, general recommendations are presented regarding the importance of monitoring the existence of ADAs in patients treated with ERT. Nevertheless, some issues remain unresolved, such as the importance of ADAs levels (and, more specifically, NAbs), the standardization of testing methods, the interpretation of results or the implication of these determinations in the therapeutic strategy.

Some consensuses specify that, in all FD patients, comprehensive routine monitoring is required to detect evidence of organ involvement and treatment response [135]. However, some unanswered questions remain regarding the progression follow-up and the assessment of clinical response. According to the authors, this is due to the heterogeneity of the disease, the long natural history of FD and the role of NAbs [26].

Currently, international antibody testing standards for assessing antibody formation in response to ERT are lacking as there are no commercially available standardized assays or protocols that have been developed to determine IgG levels. In most trials that assessed ADAs development, the manufacturing companies' own ELISA were used, and different assays were used for different forms of the enzyme. Furthermore, the analysis of ADAs neutralizing activity is an essential component of these assessments, because they allow detection of the capacity of antibodies to inhibit and/or neutralize the biological function of a given biotherapeutic in an in vitro system [136].

It is recommended that serum samples for IgG testing should be drawn prior to the first ERT infusion, every 3–6 months for the first 18 months of treatment and then every 6–12 months until two consecutive negative results are confirmed [21]. Although such scheduling may prove useful, physicians should determine the actual frequency of antibody testing according to individual patients' need for medical care and routine follow-up [21].

The recommendations of both consensuses can be summarized in the following items [20, 21]:

- ADAs should be assessed, as well as their neutralizing activity, together with lyso-Gb3 levels in patients receiving ERT [21, 38, 39].
- FD patients (mostly men with a classic phenotype) receiving ERT may experience ADAs development, and their existence should be regularly checked [20, 55, 137].
- Serum samples for IgG testing should be drawn prior to the first ERT infusion, every 3–6 months for the first 18 months of treatment and subsequently every 6 months until two consecutive negative results are confirmed [21].
- The ideal time to assess the potential existence of ADAs is right before the administration of the next dose, when the drug levels are presumably at their lowest [35].
- The potential loss of therapeutic effectiveness that is observed in some patients warrants routine measurement of ADAs in FD patients, to guide treatment adjustment [20].
- Physicians should determine the actual frequency of antibody testing according to each patient's need for medical care and routine follow-up [21, 38, 71].

Last, it must be noted that in 2016, the FDA and the National Organization for Rare Disorders (NORD) hosted a public workshop titled “Immune Responses to Enzyme Replacement Therapies: Role of Immune Tolerance Induction” to discuss the impact of ADAs on the efficacy and safety of ERTs intended to treat patients with lysosomal storage diseases [138]. Participants in the workshop included FDA staff, clinicians, scientists, patients, and industry representatives. Discussions during the workshop identifyed key knowledge gaps and future areas of research; namely [138]:

- Systematic collection of longitudinal data on immunogenicity to better understand the impact of ADAs on long-term clinical outcomes.
- Development of disease-specific biomarkers and outcome measures to assess the effect of ADAs and ITI on efficacy and safety.
- Development of consistent approaches to ADAs assays to allow comparisons of immunogenicity data across different products and disease groups, and to expedite reporting of results.
- Establishment of a system to widely share data on antibody titers following treatment with ERTs.
- Identification of components of the protein that are immunogenic so that triggers and components of the immune responses can be targeted in ITI.
- Consideration of early ITI in patients who are at risk of developing clinically relevant ADAs that have been demonstrated to worsen treatment outcomes.

## Conclusions

### Final considerations

The appearance of ADAs with ERT, particularly neutralizing (NAbs), involves a potential complication for FD patients, especially men with the classic phenotype. The impact of ADAs is fundamentally expressed in a higher IRR rate, and as for NAbs, in its clinical repercussion through a potential reduction of treatment efficacy. Although a high level of evidence is not available, we can establish that the appearance of NAbs reduces the clinical efficacy of ERT when surrogate parameters are considered, such as glomerular filtration rate, left ventricular hypertrophy (LVH) progression, or lyso-Gb3 plasma concentrations.

The presence of NAbs varies depending on the type of enzyme and the dose used, with a higher risk when higher doses are used. However, with the use of pegunigalsidase alfa, usually at the same dose as agalsidase beta, a lower ADAs rate has been documented, and they are less likely to be neutralizing in nature. Currently, the challenge is to discriminate patients that will develop antibodies, especially NAbs, and those who would benefit from switching to alternative ERT therapies.

Sound evidence is not yet available to warrant actions that should be carried out both to prevent the appearance of NAbs and their management upon detection. In fact, paradoxically, although higher doses of ERT are more immunogenic, the deleterious effect of NAbs can be blocked saturating them with high doses of ERT—in some cases different from those approved in the summary of product characteristics.

A potential preventive strategy, particularly in patients at higher risk of developing NAbs (such as males with a classic phenotype), could be the initiation of an ERT with low and gradually increasing doses, along with a lower infusion rate.

An additional and significant challenge is the limitation in quantification and interpretation of the results obtained from NAb values depending on the type of assay used. Despite everything, it may be concluded that the presence of NAbs, regardless of the analysis method used, is always clinically relevant due to their potential to block the efficacy of the drug used.

## Data Availability

Not applicable.

## Abbreviations

α-Gal A: Alpha-galactosidase A enzyme
ADA: Anti-drug antibody
ADAs: Anti-drug antibodies
BAbs: Binding antibodies
CHMP: Committee for medical products for human use
CHO: Chinese hamster ovary
CI: Confidence interval
CICs: Circulating immune complexes
COVID: Corona virus disease
CP: Cut point
CRIM: Cross reactive immunological material
CV: Coefficient of variation
DNA: Deoxyribonucleic acid
eGFR: Estimated glomerular filtration rate
ELISA: Enzyme linked immunosorbent assay
EMA: European medicines agency
ERT: Enzyme replacement therapy
FD: Fabry disease
FDA: Food and drug administration
Gb3: Globotriaosylceramide
GLA: Galactosidase alpha gene
HLA: Human Leukocyte antigen
HPC: High positive control
HRQoL: Health-related quality of life
ICs: Immune complexes
IgE: Immunoglobulin E
IgG: Immunoglobulin G
IgM: Immunoglobulin M
IRRs: Infused-related reactions
ITI: Immune tolerance induction
LBAs: Ligand binding assays
LPC: Low positive control
LVH: Left ventricular hypertrophy
Lyso-Gb3: Globotriaosylsphingosine
MAPP: MHC-associated peptide proteomics
MHCC: Major histocompatibility complex class
MoA: Mechanism of action
M6P: Mannose-6-phosphate
NAbs: Neutralizing antibodies
NORD: National organization for rare disorders
OR: Odds ratio
PD: Pharmacodynamic
PEG: Polyethylene glycol
PK: Pharmacokinetic
Pre-Abs: Pre-existing antibodies
TDM: Therapeutic drug monitoring
